# Obesity Cut-Off Points Using Prepregnancy Body Mass Index according to Cardiometabolic Conditions in Pregnancy

**DOI:** 10.1155/2023/6669700

**Published:** 2023-11-16

**Authors:** Renata O. Neves, Alexandre da S. Rocha, Bruna O. de Vargas, Daniela C. Kretzer, Salete de Matos, Marcelo Z. Goldani, Lisia von Diemen, José A. de A. Magalhães, Juliana R. Bernardi

**Affiliations:** ^1^Graduate Program in Child and Adolescent Health, Universidade Federal do Rio Grande do Sul, Porto Alegre, Brazil; ^2^Graduate Program in Health Sciences: Gynaecology and Obstetrics, Universidade Federal do Rio Grande do Sul, Porto Alegre, Brazil; ^3^Undergraduate Nutrition Course, School of Medicine, Universidade Federal do Rio Grande do Sul, Porto Alegre, Brazil; ^4^Department of Pediatric, Universidade Federal do Rio Grande do Sul, Porto Alegre, Brazil; ^5^Hospital de Clínicas de Porto Alegre, Federal University of Rio Grande do Sul, Porto Alegre, Brazil; ^6^Department of Psychiatry, Universidade Federal do Rio Grande do Sul, Porto Alegre, Brazil; ^7^Department of Obstetrics and Gynaecology, Universidade Federal do Rio Grande do Sul, Porto Alegre, Brazil; ^8^Graduate Program in Food, Nutrition and Health, Universidade Federal do Rio Grande do Sul, Medical School, Porto Alegre, Brazil

## Abstract

**Aim:**

To suggest cut-off points for body mass index (BMI) using gestational hypertension, preeclampsia, and gestational diabetes mellitus (GDM) as cardiometabolic conditions in pregnancy.

**Methods:**

In this prospective study, singleton pregnant women from the fetal medicine service of the Brazilian Unified Health System were included. The pregnancy, perinatal, and newborn data were obtained from the clinical medical records. Maternal anthropometry included an assessment of weight and height and the prepregnancy BMI evaluation categorized according to the World Health Organization cut-off points. The area under the curve and confidence interval values from receiver operator curves were generated to identify the optimal cut-off points using prepregnancy BMI with better sensitivity and specificity.

**Results:**

Data on 218 pregnancies were analyzed, with 57.9% (*n* = 124) being classified as overweight/obese, 11% (*n* = 24) with GDM, 6.9% (*n* = 15) with preeclampsia, and 11.0% (*n* = 24) with gestational hypertension. The BMI cut-off points for predicting cardiometabolic conditions were 27.52 kg/m^2^ (S: 66.7%; E: 63.8%) for women with GDM; 27.40 kg/m^2^ (S: 73.3%; E: 62.4%; S: 79.2%; E: 64.9%; S: 70.3%; E: 66.3%) for women with preeclampsia, gestational hypertension, and gestational hypertension plus preeclampsia, respectively; and 27.96 kg/m^2^ (S: 69.6%; E: 65.6%) for women with preeclampsia plus GDM.

**Conclusion:**

The findings suggest that the optimal prepregnancy BMI cut-off point is around 27 kg/m^2^ for pregnant women with maternal cardiometabolic conditions.

## 1. Introduction

The escalating prevalence of obesity is acknowledged as a worldwide public health concern that impacts individuals of all ages and genders [1]. Particularly among women of childbearing age, the increasing incidence of obesity in Brazil has significant implications for maternal health during pregnancy [2]. According to data from the Brazilian Food and Nutrition Surveillance System [3], rates of prepregnancy overweight and obesity have exhibited an upward trend, rising from 22.6% to 28.8% and from 9.8% to 19.8%, respectively, between 2008 and 2018.

The body mass index (BMI) is a widely utilized measure for assessing the nutritional status of populations, determined by measuring an individual's weight and height. The World Health Organization (WHO) has proposed BMI thresholds of 30.0 kg/m^2^ to classify individuals as obese and 25.0 kg/m^2^ for categorizing adults as overweight. These thresholds are associated with increased risks of morbidity and mortality [4]. In adults, a BMI greater than 30.0 kg/m^2^ indicates a moderate to high risk of developing comorbidities, which can be influenced by factors such as diet quality, ethnicity, and levels of physical activity [5].

Similarly, the Institute of Medicine (IOM) guidelines categorize body weight based on BMI values, classifying women as overweight (25.0-29.9 kg/m^2^) or obese (30.0 kg/m^2^), regardless of age, parity, smoking history, or ethnic background. These BMI categories have been utilized to establish recommended guidelines for gestational weight gain [6]. However, it remains uncertain whether these recommendations from the WHO and IOM adequately reflect the risk of adverse maternal and perinatal outcomes among pregnant women.

Moreover, a rise in the BMI of pregnant women is linked to an elevated risk during pregnancy, including cesarean sections (both elective and emergency), gestational diabetes, postpartum hemorrhage, preeclampsia, preterm rupture of membranes, and other associated issues. Additionally, infants born to mothers with higher BMI values face an increased likelihood of scoring below 7 on the 5-minute APGAR scale [7].

Therefore, using traditional adult BMI to predict adverse maternal or fetal outcomes among pregnant women has shown inaccuracies [8]. Consequently, this study is aimed at suggesting new BMI cut-off points based on cardiometabolic conditions in pregnancy using a low-risk outpatient sample. The objective is to identify pregnant women at risk early on, allowing them to receive adequate prenatal care, ultimately leading to improved maternal and perinatal outcomes.

## 2. Materials and Methods

### 2.1. Sample

This prospective investigation included participants from the period 2016 to 2018 at the Ultrasound Department at Murialdo Reference Health Center, which provides fetal medicine services to the Unified Health System (Sistema Único de Saúde (SUS)), in Porto Alegre, the state capital in the southernmost of Brazil.

According to the research protocol, pregnant women from the three trimesters attending a public obstetric ultrasound service were invited to participate. After obtaining informed consent, they responded to a maternal, clinical, and sociodemographic questionnaire. Cases of fetal malformations, genetic syndromes, or abortions detected by ultrasound were initially excluded.

### 2.2. Clinical Data

Baseline characteristics and pregnancy outcomes were obtained from the clinical medical records and standardized interviews by trained healthcare staff. Maternal data included age (in years), race/ethnicity (Caucasian vs. non-Caucasian), number of past pregnancies (prior live births), diagnosis of gestational hypertension, preeclampsia, and gestational diabetes mellitus (GDM).

Gestational hypertension was characterized by a systolic blood pressure (SBP) of ≥140 mmHg and/or a diastolic blood pressure (DBP) of ≥90 mmHg. Preeclampsia was diagnosed when the SBP was ≥140 mmHg or the DBP was ≥90 mmHg, or both, typically occurring after 20 weeks of gestation and often accompanied by proteinuria [9].

GDM was defined as having fasting glycemia ≥ 92 mg/dL at any point during pregnancy or as per results from an oral glucose tolerance test (with 75 grams of glucose) conducted during the second or third trimester. Diagnostic thresholds for GDM were set at ≥180 mg/dL after 60 minutes or ≥153 mg/dL after 120 minutes [10].

The gestational age at inclusion was determined based on prior fetal ultrasound assessments, using measurements such as crown-to-rump length or the last menstrual period (in weeks). Routine ultrasound was performed to assess fetal growth at the study baseline.

A secondary assessment was conducted during hospitalization for labor and delivery by reviewing medical records and examining pregnant conditions, perinatal factors, and newborn status. During this subsequent assessment, any newly diagnosed instances of GDM, gestational hypertension, or preeclampsia that had not been previously identified were included in the research. Birth weight (in grams) was classified as follows: less than 2500 grams as low birth weight, between 2500 and 2999 grams as insufficient weight, and weights equal to or above 3000 grams were considered satisfactory weights [11, 12]. Preterm birth was defined as delivered before 37^0/7^ weeks of gestation [13].

### 2.3. Maternal Anthropometric Measures

Maternal anthropometric assessment encompassed measurements of weight and height at the initial assessment. Women were advised to wear minimal clothing and abstain from footwear or any accessories like watches, bracelets, or earrings. Body weight was quantified in kilograms using a Marte® LC200-PP digital scale (São Paulo, Brazil) with a 50-gram precision. Height was measured in meters by an extensible portable stadiometer Alturexata® (Minas Gerais, Brazil).

Prepregnancy maternal weight was obtained from the prenatal record in the first weeks of pregnancy, normally before the 12th week. In cases where this information was not available, maternal report of prepregnancy weight was used. Maternal height was measured during the baseline interview.

Prepregnancy BMI (kg/m^2^) was estimated using the formula prepregnancy weight divided by the current height squared. At baseline, the BMI adult classification was underweight (BMI < 18.5 kg/m^2^), adequate weight (BMI between 18.5 and 24.9 kg/m^2^), overweight (BMI between 25.0 and 29.9 kg/m^2^), and obese (BMI ≥ 30.0 kg/m^2^), according to WHO [5, 14]. Anthropometric measurements were taken in duplicate by trained nutritionists, and the average value among the measurements was considered.

Maternal weight gain during pregnancy was deemed appropriate in accordance with the 2021 guidelines from the US Preventive Services Task Force (USPSTF), as outlined in the Behavioral Counseling Interventions for Healthy Weight and Weight Gain in Pregnancy recommendation statement published in JAMA [15]. These guidelines classify weight gain based on prepregnancy BMI as follows: 12 kg to 18 kg for those in the prepregnancy underweight category, 11 kg to 16 kg for those with a normal prepregnancy weight, 6 kg to 11 kg for individuals in the prepregnancy overweight category, and 5 kg to 9 kg for those in the prepregnancy obese category.

### 2.4. Statistical Analysis

Clinical and anthropometric data were presented encompassing both quantitative and categorical variables. A normality test was performed to evaluate the distribution of quantitative variables. Quantitative variables were summarized either using the mean and standard deviation (±SD) or the median and interquartile range (IQR) (median (p25 and p75)). Categorical variables were reported with absolute frequencies (*n*) and their respective percentages (%). To determine new BMI cut-off points during pregnancy, we generated area under the curve (AUC) values and their corresponding confidence intervals from receiver operator curves (ROC). Pregnancies with adverse metabolic outcome were used as an index to estimate the best predictive sensitivities and specificities.

Statistical analyses were performed using the Statistical Package for Social Sciences (SPSS) version 21.0 and the significance level at *p* value < 0.05.

### 2.5. Ethical Aspects

The study received approval from the Research Ethics Committee of the municipality of Porto Alegre under the reference number 2.132.090. Prior to their participation, pregnant women voluntarily provided written informed consent.

## 3. Results

Total sample included 218 pregnant women screened in the three trimesters of pregnancy. The mean (±SD) gestational age at inclusion was 19.5 (±6.8) weeks, and the median age was 25.0 (21–31) years, with 17.8% (*n* = 39) of women being 19 years old or younger.

Maternal, perinatal, and newborn characteristics are presented in Tables 1 and 2, respectively. Notably, 57.9% (*n* = 124) of women exhibited overweight or obesity based on their prepregnancy BMI. Concerning pregnancy outcomes, 11.0% (*n* = 24) experienced gestational diabetes (GDM), 6.9% (*n* = 15) had preeclampsia, and 11.0% (*n* = 24) developed gestational hypertension. The majority of newborns, specifically 64.3% (*n* = 162), had a birth weight exceeding 3000 grams.

Appropriate maternal weight gain was achieved in 58% of the sample, predominantly among pregnant women at normal and obese weight gain. Overweight group presents low adequacy regarding weight gain during pregnancy. Furthermore, among pregnant women who experienced adverse outcomes, the adequacy of weight gain was achieved in 25% of those with diabetes, 28% of those with gestational hypertension, and 14% of those with preeclampsia.

Table 3 highlights the ideal maternal BMI scores during the pregestational period based on the cardiometabolic outcome during pregnancy. It is noteworthy that the suggested values reached statistical significance in relation to the studied outcomes.

BMI cut-off point of 27.52 kg/m^2^ achieved a sensitivity of 66.7% and a specificity of 63.8% in its predictive capacity for GDM. The BMI threshold at 27.40 kg/m^2^ exhibited a sensitivity of 73.3% and a specificity of 62.4% in its predictive ability for preeclampsia. Conversely, at the same BMI threshold of 27.40 kg/m^2^, it showed a sensitivity of 79.2% and a specificity of 64.9% in predicting gestational hypertension.

When considering combined maternal health outcomes, the selected cut-off points exhibited comparable performance. Specifically, a threshold of 27.40 kg/m^2^ demonstrated a sensitivity of 70.3% and a specificity of 66.3% for the combined prediction of gestational hypertension and preeclampsia, while a threshold of 27.96 kg/m^2^ displayed a sensitivity of 69.6% and a specificity of 65.6% for the combined prediction of preeclampsia and GDM.

Suggested BMI predictive capacity threshold regarding outcomes during pregnancy is compared with traditional adult thresholds in Table 4. New threshold of 27.4 kg/m^2^ exhibits higher sensitivities and specificities compared to adult BMI cut-off points for cardiometabolic conditions during pregnancy.

## 4. Discussion

Our main finding was that the best prepregnancy BMI threshold associated with cardiometabolic conditions during pregnancy was around 27 kg/m^2^. Sensitivity, considered the most significant parameter in diagnostic evaluation, achieved an approximate rate of 80% for gestational hypertension and 73% for preeclampsia outcomes. Furthermore, the ROC curve performance achieved a high AUC for gestational hypertension using a prepregnancy BMI threshold of 27.4 kg/m^2^.

Regarding the GDM outcome, BMI threshold of 27.4 kg/m^2^ reached the lowest score among the outcomes studied with a sensitivity of 66%. However, the use of the traditional adult BMI threshold of 30 kg/m^2^ showed worse performance, reaching only 50% sensitivity in the sample. Using the same traditional BMI of 30 kg/m^2^ as a predictor of poor pregnancy outcome, an average sensitivity of 50% was found in relation to preeclampsia and gestational hypertension.

Pooled analysis of cardiometabolic outcomes shows that the new suggested BMI threshold of 27.4 kg/m^2^ has better performance in predicting preeclampsia plus gestational hypertension and the association of preeclampsia plus GDM. An average sensitivity of 70% was achieved in both outcomes. The worst predictive performance of adult BMI of 30 kg/m^2^ was achieved in the combined outcome of preeclampsia plus GDM, with a sensitivity of only 39%.

Regarding weight gain during pregnancy, our sample showed average adequacy of 58%, which is higher than the international literature [16]. Research on weight gain during pregnancy in Europe and North America reveals that ideal weight gain is achieved in only 25% to 30% of pregnant women. The majority of cases tend to exceed the limit, resulting in overweight or obesity during labor and delivery according to the adult BMI. This is partly due to limited access to nutritional counseling or obtaining foods with a lower glycemic index during pregnancy.

Selection of an appropriate BMI cut-off point for Brazilian women has been examined, mainly due to the increasing prevalence of noncommunicable diseases, including obesity [17]. A recent study carried out in Brazil, focusing on women with GDM, highlights a substantial increase in maternal obesity between the 1990s and 2010 (11.1% to 46.4%). This highlights the critical importance of prepregnancy monitoring for women wishing to become pregnant [18].

Previous studies have focused on various cutoffs for pregnant women in different populations as part of efforts to reduce chronic conditions. An illustrative case is a case-control study carried out in Iran involving 270 singleton pregnancies, conceived through assisted reproductive technology. This study identified a prepregnancy BMI of 25.4 kg/m^2^ as the threshold for an increased risk of GDM. The authors found a sensitivity of 68.9% and a specificity of 62.8% [8].

Another retrospective cohort study that reviewed 11,494 medical records suggested that a lower prepregnancy BMI cutoff of 25 kg/m^2^ to define obesity might be appropriate for pregnant women in southern China. The authors suggest that this approach would better predict adverse obstetric and perinatal outcomes. Unlike the analysis in this article, the authors found that the prevalence of obesity was 7.2% (BMI ≥ 25 kg/m^2^), due to the relatively lean population [19].

A recent study of 11,136 pregnant women in China focused on pregnancy outcomes and found a linear relationship between poor outcome and increased prepregnancy adult BMI. The authors evaluated gestational hypertension with or without preeclampsia, GDM, cesarean section, postpartum hemorrhage, small-for-gestational-age newborns, and macrosomia. It was suggested that the ideal prepregnancy BMI range was between 18.5 and 22.9 kg/m^2^, with the cut-off point for overweight being 23.0 kg/m^2^ and the cut-off point for obesity being 28.0 kg/m^2^ [20].

Adult BMI score compatible with obesity is considered a strong predictor of general mortality among prospective epidemiological studies [21, 22]. In the context of pregnancy, the application of adult BMI thresholds is constrained, emphasizing that adult scores may result in low predictive sensitivity for typical adverse pregnancy outcomes [6].

In our study, most women were classified as overweight/obese prior to pregnancy, according to WHO criteria. These findings align with the global increase in overweight and obesity, which has reached epidemic levels in several countries [23]. In Brazil, a recent female cohort with 19,931 participants found that the prevalence of overweight and obesity more than doubled over the 33-year span, from 22.1% (1982) to 47.0% (2015) [24].

A systematic review and meta-analysis of 45 studies revealed that prepregnancy overweight/obesity status increases the risk of the newborn large for gestational age, macrosomia, and subsequent offspring at risk of future overweight/obesity [25]. Previous studies have shown that pregnancy obesity represents a risk factor for GDM [26], preeclampsia [26, 27], macrosomia [27–29], and cesarean section [27, 28].

Among the new and more accurate techniques for predicting adverse pregnancy outcomes, the measurement of maternal visceral adiposity using ultrasonography is becoming promising. GDM outcome with measurements greater than 4.7 cm in the Armellini region (distance from the linea alba to the aortic cross in the maternal supraumbilical region in the first 20 weeks) showed a significant odds ratio of 16.9 [30]. A similar study found a significant odds ratio of 14.4 among Armellini measurement greater than 4.5 cm in nonobese prepregnant women [31]. Regarding the outcomes of preeclampsia and premature birth with preeclampsia, Armellini region measurement above 5.2 cm between 11 and 14 weeks of gestation showed a significant relative risk of 3.1 and 16.9, respectively, even after control of concomitant pregnancy conditions [32].

The study's main strength was the disease search at baseline and during the hospitalization process for labor and delivery, resulting in a GDM, gestational hypertension, and preeclampsia double check. Furthermore, the study focused in low-risk pregnant women, which simulates the majority of outpatient care during pregnancy. Here, simple maternal anthropometric measurements can become tools for decision-making in clinical practice routine.

Limitations during the study unfortunately existed, including self-reported prepregnancy weight, reported by some patients when weight information was not available in first-trimester prenatal records. However, several studies demonstrated that using self-reported weight in pregnant women and young adults is valid [33, 34]. Furthermore, total weight gain during pregnancy was obtained in approximately 55% of the sample. This fact is due to the lack of routine measurement of maternal weight during hospitalization to labor and delivery.

## 5. Conclusions

In summary, the present study suggests that the use of adult BMI thresholds among pregnant women may not be universally applicable to all scenarios. Instead, adopting 27 kg/m^2^ as a cut-off point threshold appears to be more effective in predicting adverse pregnancy-related outcomes, achieving a better balance between sensitivity and specificity. This allows for early identification of pregnant women at risk for common metabolic outcomes of pregnancy with superior accuracy. Ultimately, early implementation of rigorous clinical and nutritional care will lead to better maternal and perinatal outcomes.

## Figures and Tables

**Table 1:** Maternal and pregnancy characteristics (*n* = 218).

Variables	Cut-off points	Frequency, *n* (%) or median (IR)
Maternal age (years)	—	25 (21; 31)

Caucasian^∗^ (*n* = 217)	—	117 (53.9)

Parity^∗^ (*n* = 210)	—	2 (1; 3)

Prepregnancy BMI^∗^ (*n* = 214)	<25 kg/m^2^	90 (42.1%)
	25.0-29.9 kg/m^2^	64 (29.9%)
	≥30 kg/m^2^	60 (28.0%)

Maternal adequate weight gain (*n* = 139)^∗^	Underweight/normal	43 (68%)
	Overweight	17 (39%)
	Obese	21 (63%)

GDM	—	24 (11.0%)

Preeclampsia	—	15 (6.9%)

Gestational hypertension	—	24 (11.0%)

BMI: body mass index; GDM: gestational diabetes mellitus; IR: interquartile range. ^∗^Totals may not add up to 218 because of missing values.

**Table 2:** Perinatal and newborn characteristics (*n* = 218).

Variables	Cut-off points	Frequency, *n* (%)
Cesarean section	—	60 (27.5%)

Labor (weeks)	<37	16 (7.6%)

Newborn weight (grams)	<2.500	13 (6.0%)
	2.500-2.999	43 (19.7%)
	≥3000	162 (64.3%)

Meconium during labor^∗^ (*n* = 213)	—	36 (16.9%)

^∗^Totals may not add up to 218 because of missing values.

**Table 3:** Prepregnancy body mass index estimated according to cardiometabolic conditions in pregnancy.

Cardiometabolic conditions	AUC (CI)^∗^	*p* value	Prepregnancy BMI (sensibility-specificity)
GDM (*n* = 24)	0.682 (0.565-0.799)	0.004	27.52 kg/m^2^ (S: 66.7%; E: 63.8%)
Preeclampsia (*n* = 15)	0.695 (0.560-0.831)	0.012	27.40 kg/m^2^ (S: 73.3%; E: 62.4%)
Gestational hypertension (*n* = 24)	0.708 (0.590-0.826)	0.001	27.40 kg/m^2^ (S: 79.2%; E: 64.9%)
Gestational hypertension+preeclampsia (*n* = 37)	0.710 (0.616-0.805)	<0.001	27.40 kg/m^2^ (S: 70.3%; E: 66.3%)
Preeclampsia+GDM (*n* = 23)	0.632 (0.510-0.753)	0.039	27.96 kg/m^2^ (S: 69.6%; E: 65.6%)

^∗^Area under the curve (AUC) from the receiver operator curve (ROC); BMI: body mass index; CI: confidence interval; GDM: gestational diabetes mellitus.

**Table 4:** Metabolic outcomes predictive capacity (adult × pregnant BMI threshold).

Cardiometabolic conditions	Cut-off point prepregnancy BMI (kg/m^2^)	Sensibility (%)	Specificity (%)
GDM (*n* = 24)	25.00	75.0	45.7
	27.52	66.7	63.8
	30.00	50.0	75.5

Preeclampsia (*n* = 15)	25.00	73.3	44.7
	27.40	73.3	62.4
	30.00	53.3	74.6

Gestational hypertension (*n* = 24)	25.00	79.2	46.3
	27.40	79.2	64.9
	30.00	54.2	76.1

Gestational hypertension+preeclampsia (*n* = 37)	25.00	75.7	47.4
	27.40	70.3	66.3
	30.00	51.4	78.3

Preeclampsia+GDM (*n* = 23)	25.00	69.6	45.0
	27.96	69.6	65.6
	30.00	39.1	74.1

BMI: body mass index; GDM: gestational diabetes mellitus.

## Data Availability

Full database was uploaded at Phisionet Journal Repository under numbers doi:10.13026/p729-7p53 and doi:10.13026/zedg-p783.
